# Ppe.XapF: High throughput KASP assays to identify fruit response to *Xanthomonas arboricola* pv. *pruni* (*Xap*) in peach

**DOI:** 10.1371/journal.pone.0264543

**Published:** 2022-02-25

**Authors:** Margaret B. Fleming, Texanna Miller, Wanfang Fu, Zhigang Li, Ksenija Gasic, Christopher Saski

**Affiliations:** 1 Department of Plant Biology, Michigan State University, East Lansing, MI, United States of America; 2 Department of Plant and Environmental Sciences, Clemson University, Clemson, SC, United States of America; USDA-ARS Southern Regional Research Center, UNITED STATES

## Abstract

Bacterial spot, caused by *Xanthomonas arboricola* pv. *pruni* (*Xap*), is a serious peach disease with symptoms that traverse severe defoliation and black surface pitting, cracking or blemishes on peach fruit with global economic impacts. A management option for control and meeting consumer demand for chemical-free, environmentally friendly fruit production is the development of resistant or tolerant cultivars. We developed simple, accurate, and efficient DNA assays (Ppe.XapF) based on SNP genotyping with KASP technology to quickly test for bacterial spot resistance alleles in peach fruit that allows breeders to cull seedlings at the greenhouse stage. The objective of this research was to validate newly developed DNA tests that target the two major QTLs for fruit resistance in peach with diagnostic utility in predicting fruit response to bacterial spot infection. Our study confirms that with only two Ppe.XapF DNA tests, Ppe.XapF1-1 and Ppe.XapF6-2, individuals carrying susceptible alleles can be identified. Use of these efficient and accurate Ppe.XapF KASP tests resulted in 44% reduction in seedling planting rate in the Clemson University peach breeding program.

## Introduction

Peach (*Prunus persica* L. Batsch) belongs to the *Prunus* genus of the Rosaceae family and is one of the most economically important fruit tree crops worldwide [1]. It is extensively grown throughout the temperate zone for its delicious and healthy fruit [2, 3]. China is the world’s largest producer of peaches and nectarines, followed by Spain, Italy, Greece, Turkey and the U.S with a global annual production of ~25 million tons [4].

One of the major obstacles in growing peaches and nectarines worldwide is fruit susceptibility to diseases. No peach cultivar resistant to major peach diseases currently occupies any substantial U.S. market share, and despite the hundreds of existing peach cultivars used for fresh market, there is continuing need to develop new peach cultivars as the requirements of the industry and preferences of consumers change [5]. Diseases such as bacterial spot create an extra pressure on breeding programs to incorporate high genetic tolerance or resistance into new high-quality varieties for three main reasons: existing cultivars are not resistant [6–8]; chemical spraying does not provide adequate protection during pathogen-favorable years [6, 7]; and the existing pathogen continues to evolve into new races that are resistant to active compounds used in chemical spraying programs [8].

Bacterial spot, caused by *Xanthomonas arboricola* pv. *pruni* (*Xap*), is a serious disease that affects nearly all cultivated *Prunus* species and their hybrids [7, 9]. The most severe infections have been reported on Japanese plum (*P*. *salicina*), Korean cherry (*P*. *japonica*) and plum hybrids, as well as on peach and nectarines (*P*. *persica*) and their hybrids [10]. Disease etiology of bacterial spot on the peach tree includes fruit spots, leaf spots, and twig cankers. Symptoms on the peach fruit include pitting, cracking, gumming, and water-soaked tissue, which in turn can increase the susceptibility of the fruit to other fungal infections, such as Rhizopus and brown rot. Eventually, severe leaf spot infections can cause early tree defoliation, resulting in reduced vigor and winter hardiness [10]. Conventional methods of control include the use of copper-based compounds or antibacterial sprays such as oxytetracycline, but these are only effective in years with low to medium disease pressure. There is also concern with excessive antibiotic use and heavy metal accumulation in the environment, which has directed the focus on durable genetic resistance as a long-term solution.

Peach is highly susceptible to *Xap* and most peach cultivars exhibit a high degree of variation in disease susceptibility [11]. The most effective control for *Xap* is by incorporation of resistant alleles into the host plant through breeding. *Xap*-tolerance was introgressed from ‘Elberta’ into the popular commercial cultivar J.H. Hale [12], resulting in a few resistant cultivars such as Clayton and Candor [13]. Unfortunately, many resistant cultivars, such as these, lack desirable fruit and marketing characteristics [12]. Most cultivars currently in production are susceptible to *Xap* with origins that trace back to the high-quality cultivar O’Henry, which is highly susceptible to both leaf and fruit *Xap* infections. Therefore, a need is still present to introgress *Xap* resistance into high-quality varieties that span the ripening season and are adaptable to various environmental conditions.

Initial success in developing bacterial spot resistant peach cultivars suggested that resistance might be conferred by only a few dominant genes [14]. Inconsistent levels of leaf and fruit resistance in the same peach cultivar also indicated involvement of separate genetic factors in leaf and fruit resistance [8, 15]. Several controlling loci in the peach genome conferring quantitative resistance have been reported in bi-parental mapping population [15]. Out of 14 quantitative trait loci (QTLs) detected, four were deemed to have major effects: one on leaf, one on both leaf and fruit, and two on fruit response to bacterial spot infection in peach. Two QTLs with the largest effects (43.6% each) on bacterial spot resistance in peach fruit were detected, one each on chromosome (ch) 1 (*Xap*.*Pp*.*OC-1*.*2*; 12.9–14.9 Mb; 23–43.5 cM) and ch 6 (*Xap*.*Pp*.*OC-6*.1; 22.2–22.3 Mb; 3.9–4.7 cM) [15] (www.rosaceae.org). Using the peach 9K SNP array genotype data, obtained from peach germplasm relevant for the four public U.S. peach breeding programs [16, 17], the *Xap*.*Pp*.*OC-1*.*2* and *Xap*.*Pp*.*OC-6*.*1* haploblocks were found to include five and six haplotypes/alleles, with four and eight SNPs, respectively [18]. Allele frequency and effect analyses revealed four widely distributed alleles associated with fruit disease phenotypes: resistant (R)1, R2, susceptible (S), and intermediate (I) [18]. The effect of a fifth allele, present only in almond (alm) and peach × almond hybrids, was not determined [18]. Furthermore, the associations between allele and disease phenotype, observed in the four peach breeding programs involved in the RosBREED project, suggest a possible epistatic effect between the two QTLs, with R alleles on ch 6 providing higher tolerance than those on ch 1 (unpublished data). Using this information, simple sequence repeat (SSR) diagnostic tests for fruit response to bacterial spot infection in peach, Ppe-Xap-LG1 and Ppe-Xap-LG6, were developed within the RosBREED project (www.rosbreed.org) [19]. However, the tests could not distinguish between all *Xap*.*PpOC-1*.*2* and *Xap*.*Pp*.*OC-6*.*1* alleles and only had partial accuracy and agreement with 9K SNP data.

There is great urgency among the peach breeding communities to develop more efficient ways to incorporate disease resistance with high fruit quality and productivity into newly developed peach varieties. Despite vast genetic and genomic resources for peach [20, 21], most efforts stop after revealing loci in the peach genome associated with traits of interest, and rarely follow through to develop tools for breeders to use for DNA-informed breeding (marker-assisted breeding) [22]. A high throughput DNA test to predict bacterial spot phenotypes in breeding germplasm and segregating progeny at the seedling stage can significantly reduce screening and selection costs, while increasing breeding program efficiency. Kompetitive Allele Specific PCR (KASP) assays are a rapid and robust technique to genotype SNPs of interest [23–25]. The objective of this study was to develop and validate a rapid and unambiguous DNA test, using the previously identified associations between disease phenotypes and SNP alleles within the *Xap*.*Pp*.*OC-1*.*2* and *Xap*.*Pp*.*OC-6*.*1* QTLs, that can be used in peach breeding for accurate and routine prediction of bacterial spot fruit response.

## Material and methods

### Plant material

The peach material (84 cultivars and advanced selections, Table 2) used for development and validation of the Ppe.XapF DNA test (Table 2), is part of the *Prunus* germplasm collection maintained at the Clemson University Musser Fruit Research Center, Oconee County in Seneca, SC (Latitude:34.639038, Longitude: -82.935244, Altitude 210 msl), under standard commercial practices for irrigation, fertilization, and pest and disease control. The trees were at least 5 years old, grafted on Guardian® rootstock, planted in duplicate at 1.5 m × 4 m spacing and trained to a perpendicular V system. The material selected for this study represents a mixture of new and old peach cultivars, including important founders, breeding parents, recently released cultivars, and advanced selections developed or used in the Clemson University peach breeding (CUPB) program. Peach material was also selected based on the availability of genotypic [17] and phenotypic [12] data. The cultivars planted in the Clemson University collection and included in this study are obtained from the Adams County Nursery (Aspers, PA, USA). In addition, 3,440 seedlings generated from crosses in the CUPB program were used to test the Ppe.XapF KASP assay’s performance with crude DNA extracts [26].

Bacterial spot response of the peach cultivars/breeding material used for assay validation were identified from literature [12] or plant patent information (United State Plant Patent patft.uspto.gov) when available. Bacterial spot responses used in cultivar descriptions and described in Okie [12] were converted to values corresponding to bacterial spot infection levels described in Yang et al. [15] and used in Gasic et al. [18]: highly resistant = 0; resistant = 1; moderately resistant = 2; moderately susceptible = 3; susceptible = 4; and highly susceptible = 5.

### DNA extraction

Development and validation of the Ppe.XapF KASP assay used high-quality DNA extracted from young leaf tissue following a protocol modified from Edge-Garza *et al*. [27]. Approximately 100 mg of fresh leaf tissue was collected per well of a 96-well plate (Ab-Gene AB-0661) and a stainless-steel bead was added. The tissue was lyophilized in a LABCONCO LYPH-LOCK 6 freeze dryer and ground to a fine powder using a Geno-grinder (SPEX). Extraction buffer (100 mM Tris-HCl pH 8.0, 50 mM EDTA pH 8.0, 500 mM NaCl, 1% SDS, 1% PVP40, 500 μg/mL proteinase K, and 1% DTT) was prepared prior to extraction and heated to 65°C before adding 0.5 mL to each well of the plate. The plate was sealed and inverted to mix. The plate was incubated at 65°C for 30 min with occasional agitation, then cooled at -20°C for 15 min. After cooling, 250 μL of cold (4°C) 6M ammonium acetate was added to each well and the plate was resealed, inverted, and returned to -20°C for an additional 15 min. The plate was centrifuged in a swing-bucket bench top centrifuge for 20 min at 1,976 g at 4°C. After centrifugation, ~400 μL of supernatant was transferred from each well to a combination filter/receiver plate (Pall Filter# 8130; Ab-gene receiver AB-0859) and centrifuged at 1,147 g for 7 min. The filter was discarded and 240 μL of chilled isopropanol was added to each well. The plate was sealed, mixed and placed at 4°C overnight to precipitate DNA. The next day the plate was centrifuged at 1,792 g for 30 min at 4°C and decanted. Pelleted gDNA in each well was washed twice with 450 μL of 70% ethanol and resuspended in 50 μL of DNase/RNase free water (Gibco). DNA was RNase treated with 1 U of RNase A (ThermoFisher) for 1 hour at 37°C.

Crude DNA extraction from seedlings either in the greenhouse or in the field followed the protocol of Noh *et al*. [26] in a 96-well plate format, using one 3 mm leaf disc per plant per well. Plates were kept on ice during tissue collection. Directly following tissue collection, 50 μL of freshly prepared buffer A (100 mM NaOH, 2% Tween 20) was added to each well. The plate was sealed with foil tape, centrifuged at 1,792 g for 2 min, and heated at 95°C for 10 min. An equal volume (50 μL) of buffer B (100 mM Tris-HCl pH 8, 2 mM EDTA) was added to each well. The plate was centrifuged at 1,792 g for 2 min, sealed with fresh foil tape, and stored at 4°C overnight. The next day each sample was diluted with addition of 100 μl of distilled water, sealed with fresh foil tape and stored for a week at 4°C or -20°C until use.

DNA concentration after either extraction protocol was checked on a subset of samples with a Nanodrop spectrophotometer. Observed concentrations ranged between 900 and 1000 ng μL^-1^ for standard extractions, or between 180 and 400 ng μL^-1^ for crude extractions. DNA was diluted 200× from all standard extractions and 40× from all crude extractions in nuclease-free water to achieve the recommended concentration for KASP assays of 5 ng μL^-1^. Crude DNA extraction, dilution and PCR plate set up for both real time and endpoint reactions for both germplasm and seedling samples was conducted with the OT-2 robot from Opentrons (opentrons.com).

### Primer design

For both Xap haploblocks, haplotypes/alleles were previously associated with varying levels of *Xap* fruit response and used in this study for SNP selection [18]. To distinguish the five unique alleles detected on ch 1, all four SNPs in *Xap*.*Pp*.*OC-1*.*2* are required, while for the six alleles on ch 6, four of the eight SNPs in *Xap*.*Pp*.*OC-6*.*1* are sufficient (Table 1) [18]. Primer sets for KASP assays were designed for seven of these SNPs, identifying the presence of an “A” (A or T, assigned to the FAM fluorophore) or a “B” nucleotide (G or C, assigned to the HEX fluorophore) (S1 Table). Primers were designed to meet the following criteria: GC content between 30–55%; Tm of ~64°C ± 2; 21–30 bp long; product size of 50–100 bp; secondary structure more positive than -9 kca/mole; no more than four di-nucleotides; no more than 4 or 5 identical nucleotides in a row; no more than 3 Gs and/or Cs in the last 5 bp of the primer. Primers were not designed for SNP_IGA_680889 on ch 6, as the surrounding sequence could not meet the design criteria.

**Table 1:** Ppe.XapF SNPs, alleles [18], 9K IPSC peach array [17] codes and their associated phenotypes.

QTLs	SNPs	Nucleotides					9K IPSC codes						Ppe.XapF
		S	R1	R2	I	alm	S	R1	R2	I	alm	-	
*Xap*.*Pp*.*OC-1*.*2*	SNP_IGA_39717	T	C	C	C	C	A	B	B	B	B		**1–1**
	SNP_IGA_40295	A	G	G	G	A	A	B	B	B	A		1–2
	SNP_IGA_43384	T	T	C	T	T	A	A	B	A	A		1–3
	SNP_IGA_46754	G	A	G	G	G	B	A	B	B	B		1–4
*Xap*.*Pp*.*OC-6*.*1*	SNP_IGA_680615	T	G	G	T	T							+
	SNP_IGA_680882	C	T	C	C	C							+
	SNP_IGA_680889*	C	C	T	T	T	B	B	A	A	A	A	6–1
	SNP_IGA_680909	T	C	C	T	C	A	B	B	A	B	B	**6–2**
	SNP_IGA_680953	A	G	A	G	G	A	B	A	B	B	B	6–3
	SNP_IGA_681081	G	G	A	G	A	B	B	A	B	A	B	6–4
	SNP_IGA_681113	T	T	G	T	G							+
	SNP_IGA_681119	G	G	A	G	A							+

S–susceptible; R1 and R2 –resistant; I–intermediate; alm–allele observed in almond;—allele with unknown bacterial spot phenotype

*SNP that could not be used in KASP. Bolded SNPs used for culling susceptible seedlings. +–redundant SNPs.

### KASP assay

A primer master mix of both forward primers and the reverse primer for a single SNP assay was assembled as follows, after resuspending primers in nuclease-free water at 100 μM: 18 μL of each forward primer and 45 μL of the reverse primer were combined with 69 μL of 10 mM Tris-HCl, pH 8.0. The reaction mixture for each assay included 432 μL of 2× PACE™2.0 genotyping master mix with no ROX (3CR Bioscience, Harlow, Essex, UK) (which includes polymerase, dNTPs, buffer, and HEX- and FAM-tagged oligonucleotides) and 11.88 μL of the appropriate primer master mix. Genotyping assays used 5 μL of reaction mixture and either 5 μL of water (no-template control) or 5 μL of genomic DNA at approximately 5 ng/μL. Three replicates of no-template controls and positive controls for AA, AB, and BB genotypes for the tested SNPs were always included, along with unreplicated samples of unknown genotype (“unknowns”). Positive controls were chosen from 18 accessions previously genotyped with the peach 9K SNP array [17] as part of the Peach Crop Reference Set [28] within the RosBREED project (www.rosbreed.org) (Table 2). A total of 3,440 seedlings were organized in ~40 96-well plates using the OT-2 robot, with all control samples in row A at the 2.5 ng/μL DNA concentration (S2 Table). As control sample DNA was of a high quality, we reduced its concentration from the recommended 5 to 2.5 ng/μL to adjust fluorescence levels between controls and the crude DNA.

**Table 2:** Peach germplasm used for development and validation of the Ppe.XapF DNA test. Ppe.XapF genotypes predicted with KASP and 9K IPSC SNP array.

Name	Type	Disease Index	Ppe.XapF			
			KASP		Array	
			F1	F6	F1	F6
ArcticBelle	cultivar		R1|R1	S|S		
ArcticBlaze	cultivar		R1|S	R1|S		
ArcticGold	cultivar		R1|R1	R1|S		
ArcticPride	cultivar		R1|S	S|S		
ArcticStar	cultivar		R1|R1	S|S		
ArcticSweet	cultivar		R1|S	R1|S		
Arrington	cultivar	2	S|S	R2|R2	S|S	R2|R2
Autumnflame	cultivar		R1|S	S|S		
Autumnprince	cultivar	3	R1|S	S|S		
Autumnred	cultivar		S|S	S|S		
Blazeprince	cultivar	5	S|S	S|S	S|S	S|S
Blazingstar	cultivar	1	R1|S	R2|S	R1|S	R2|S
Bounty	cultivar	2	R1|R1	R2|S	R1|R1	R2|S
Bradley	cultivar	2	S|S	R2|R2	S|S	R2|R2
BY00P4945	breeding material		S|S	R1|R1		
BY00P6346u	breeding material	2	R1|R1	R1|S	R1|R1	R1|S
BY01P9169c	breeding material		I|I	R1|R2		
BY01P9239	breeding material		S|S	S|S		
**BY07n3500**	breeding material	2	**I**|S	R2|S	**R1**|S	R2|S
BY99P4366	breeding material		S|S	R1|R1		
Caroking	cultivar		R1|R1	R2|S	R1|R1	R2|S
Carored	cultivar		S|S	R2|S	S|S	R2|S
CaryMac	cultivar		R1|R1	R2|R2		
China Pearl	cultivar	3	I|-	R2|S	I|-	R2|S
Chinese cling	cultivar	2	R1|S	R2|I	R1|S	R2|I
Clayton	cultivar	0	R1|S	R1|R2	R1|S	R1|R2
Clemson Lady	cultivar		R1|S	S|S		
Contender	cultivar	2	R1|S	S|S	R1|S	S|S
Coronet	cultivar	4	R1|S	S|S		
Crimson Lady	cultivar		R1|S	S|S	R1|S	S|S
CVN-1	cultivar		S|S	S|S		
Dixired	cultivar	2	R1|S	R2|S		
Elberta	cultivar	2	R1|S	R1|S	R1|S	R1|S
Empress	cultivar	4	S|alm	R2|S		
Fireprince	cultivar	2	R1|S	S|S	R1|S	S|S
Flameprince	cultivar	2	R1|S	R2|S	R1|S	R2|S
Flavorich	cultivar		R1|alm	S|S		
*FlavorTop*	cultivar	4	R1|S	R1|S		
GlacierWhite	cultivar		S|S	S|S		
Glenglo	cultivar		R1|R1	R2|R2		
Goldcrest	cultivar	2	S|S	R2|S	S|S	R2|S
Hakuto	cultivar		S|S	S|S	S|S	S|S
Harrow Diamond	cultivar	2	S|S	R2|R2		
*Harvester*	cultivar	2	S|S	S|S	S|S	S|S
Honey Blaze	cultivar		R1|R1	R2|S		
Intrepid	cultivar		S|S	R1|R2	S|S	R1|R2
Jayhaven	cultivar	2	S|S	R2|R2		
Joanna Sweet	cultivar		R1|S	S|S		
Julyprince	cultivar	2	R1|S	R1|S	R1|S	R1|S
Loring	cultivar	0	R1|R1	R2|R2	R1|R1	R2|R2
O’Henry	cultivar	5	S|S	S|S	S|S	S|S
Raritan Rose	cultivar	2	R1|I	R1|R1	R1|I	R1|R1
Redhaven	cultivar	2	R1|S	R2|S		
**Reliance**	cultivar	1	**-|-**	R2|S	I|I	R2|R2
Rich Joy	cultivar	2	R1|S	R2|S	R1|S	R2|S
SC08_02_012	breeding material		S|S	S|S	S|S	
SC08_09_006	breeding material		R1|R1	S|S	R1|R1	
*SC08_13_001*	breeding material	0	R1|S	R2|S	R1|S	
SC08_16_005	breeding material		S|S	S|S	S|S	
SC08_16_070	breeding material	2	R1|S	R1|S	R1|S	
SC08_17_059	breeding material		R1|S	R2|R2	R1|S	
Scarletprince	cultivar		R1|S	R2|S	R1|S	
September Snow	cultivar		R1|S	S|S	R1|S	
Snowbrite	cultivar		R1|S	S|S	R1|S	
Snowprince	cultivar		S|S	S|S	S|S	
Stark Saturn	cultivar		R1|S	R1|R1	R1|S	
Sugar Lady	cultivar		R1|S	S|S	R1|S	
Summergold	cultivar		R1|S	R2|S	R1|S	
Summerprince	cultivar	2	R1|S	R2|R2	R1|S	R2|R2
Summer Sweet	cultivar		S|S	S|S		
Sunbrite	cultivar		S|S	S|S		
Suncrest	cultivar		S|S	S|S		
SuziQ	cultivar		S|alm	R2|S		
Sweet Blaze	cultivar		R1|S	S|S		
Sweet Dream	cultivar	3	R1|R1	S|S		
Topaz	cultivar		R1|R1	R2|R2		
UF Gold	cultivar		R1|S	R2|R2	R1|S	R2|R2
Vulcan	cultivar		R1|S	R2|R2		
Westbrook	cultivar	1	R1|S	R2|R2	R1|S	R2|R2
White Lady	cultivar		S|S	R2|S		
Wild Rose	cultivar		R1|I	R2|S		
*Winblo*	cultivar	2	S|S	S|S	S|S	S|S
Yukon King	cultivar		R1|R1	S|S		
Zephyr	cultivar		R1|R1	S|S		

Disease index is based on the following scale: 0 –highly resistant; 1 –resistant; 2 –moderately resistant; 3 –moderately susceptible; 4 –susceptible; 5 –highly susceptible [15]. Bolded accessions are instances where KASP and array data disagree. Italicized accessions are instances where the reported phenotype disagrees with the phenotype predicted by the alleles. Underlined accessions are used as controls in KASP assays.

Reactions were performed in a Bio-Rad CFX Connect Real-Time PCR Detection System using the following program for all SNPs except Ppe.XapF1-3 and F6-2: 15 min at 95°C (activation), followed by 10 touchdown cycles of 94°C for 20s (denaturing), 61-55°C for 60s (dropping 0.6°C per cycle, for annealing and elongation), followed by 40 cycles of 94°C for 20 s, 55°C for 60s, 23°C for 30s (for accurate plate reading). The Ppe.XapF1-3 and Ppe.XapF6-2 required higher temperatures for annealing/elongation (58°C for XapF1-3, 57°C for XapF6-2) to generate clearly separated genotype clusters, and consequently used a smaller temperature decrement during the 10 touchdown PCR cycles. For each plate, the cycle used for genotype assignment was chosen to maximize separation between genotype clusters and minimize background amplification, usually between cycles 22–28 of the 40-cycle period. Endpoint PCR reactions, omitting the step of 23°C for 30s and plate reading, were performed on Bio-Rad T100 thermal cyclers to assay Ppe.XapF1-1 and Ppe.XapF6-2 using 25 cycles, as identified from real-time PCR. High-quality DNA from previously genotyped samples was used first to validate the method, followed by crude DNA extracts from seedlings. Endpoint assays were read on the Bio-Rad Real-Time machine using Bio-Rad CFX Maestro™ software.

### Data analysis

To account for differences in fluorescence values between fluorophores and assays, HEX and FAM relative fluorescence units for each sample were transformed to reflect the percentage of the maximum fluorescence for each fluorophore within a plate

%fluorescence=100×(samplefluorescence−minimumfluorescence)/(maximumfluorescence−minimumfluorescence)

To assign genotypes to unknowns, the difference between % HEX fluorescence and % FAM fluorescence (“delta”) was calculated. Heterozygotes are expected to have approximately equal HEX and FAM fluorescence, yielding delta values close to 0. As primers were designed so that all A alleles were assigned to FAM fluorescence and all B alleles to HEX fluorescence, BB genotypes (high HEX fluorescence, low FAM fluorescence) are expected to yield positive delta values and AA genotypes (low HEX fluorescence, high FAM fluorescence) are expected to yield negative delta values. The exact cutoffs for genotype assignment were determined manually for each assay. Any samples with fluorescence values < 20% for both fluorophores were considered to have failed to amplify (S3 Table).

Genotyping just one SNP on each chromosome (F1-1 and F6-2) can identify susceptible alleles, and for both SNPs the A allele (A|T) indicates the S or S|I phenotype, respectively. For high-throughput seedling screening, endpoint assays with Ppe.XapF1-1 and F6-2 were used: seedlings with A allele frequency ≥ 3 (i.e., homozygous A for one SNP and heterozygous for the other) were culled (S4 Table).

## Results

### Genotyping results from seven SNPs

Five alleles on chromosome 1, in *Xap*.*Pp*.*OC-1*.*2*, and six alleles on chromosome six, in *Xap*.*Pp*.*OC-6*.*1*, can be uniquely identified with four SNPs each (Table 1) [17, 18]. Alleles and their associated bacterial spot phenotypes [susceptible (S), almond (alm), intermediate (I), resistant-1 (R1) and resistant-2 (R2)] are presented in Table 1. One SNP, which differentiates between the heterozygous alleles S|alm and I|R2 on chromosome 6, was not suitable for a KASP assay.

**Fig 1:**
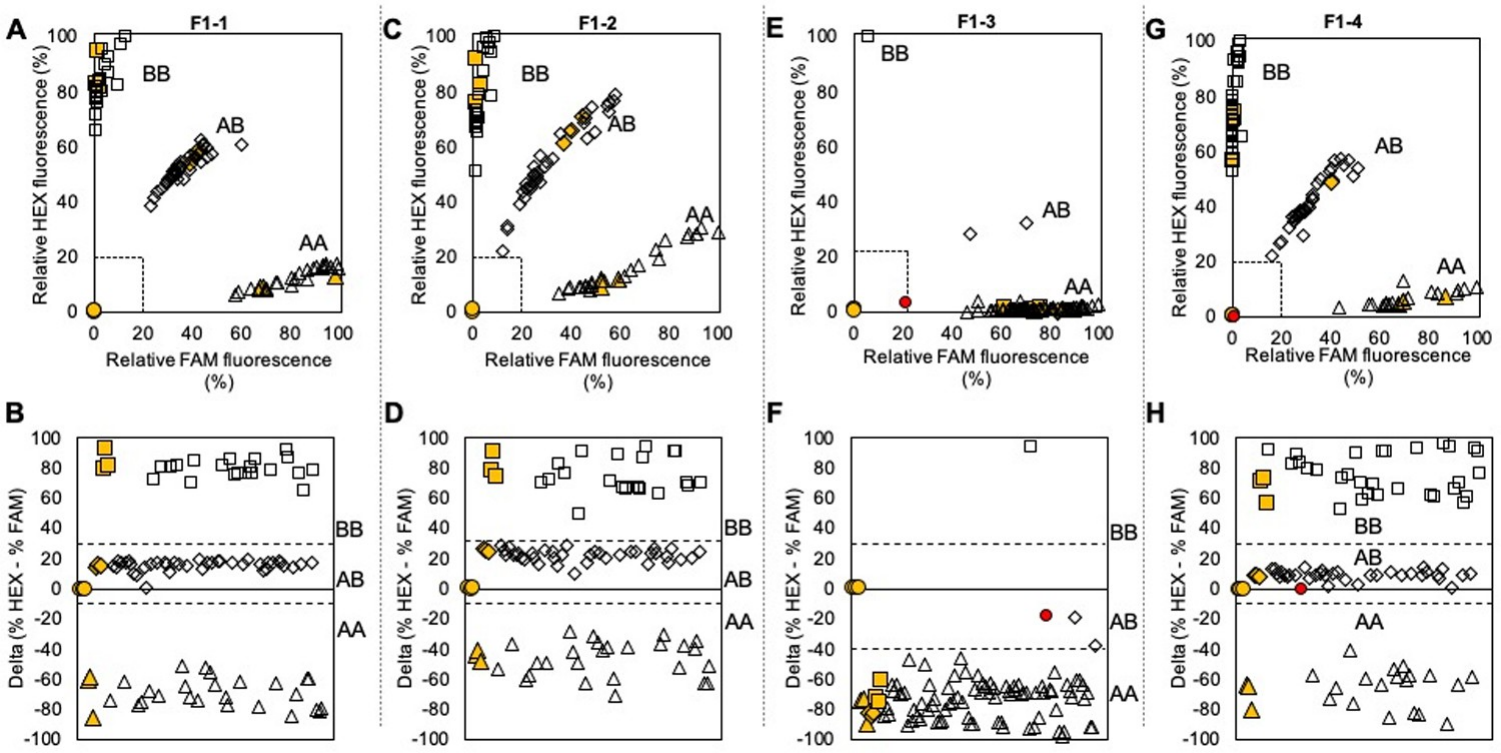
Results from KASP assay for SNPs on Ppe.XapF1 using clean DNA extraction and previously genotyped samples. Solid yellow shapes indicate positive controls, solid red indicates an unknown that failed to amplify, and empty shapes indicate unknowns, with the shape indicating the assigned genotype as follows: circle = no template/no amplification, triangle = AA, diamond = AB, square = BB. Dashed lines in plots in the top row indicate boundaries for no amplification (% fluorescence < ~20% for both fluorophores). Dashed lines in plots in the bottom row indicate delta (% HEX—% FAM) values separating heterozygous and homozygous genotypes, adjusted to reflect assay conditions. **A,B** = F1-1; **C,D** = F1-2; **E,F** = F1-3; **G,H** = F1-4.

**Fig 2:**
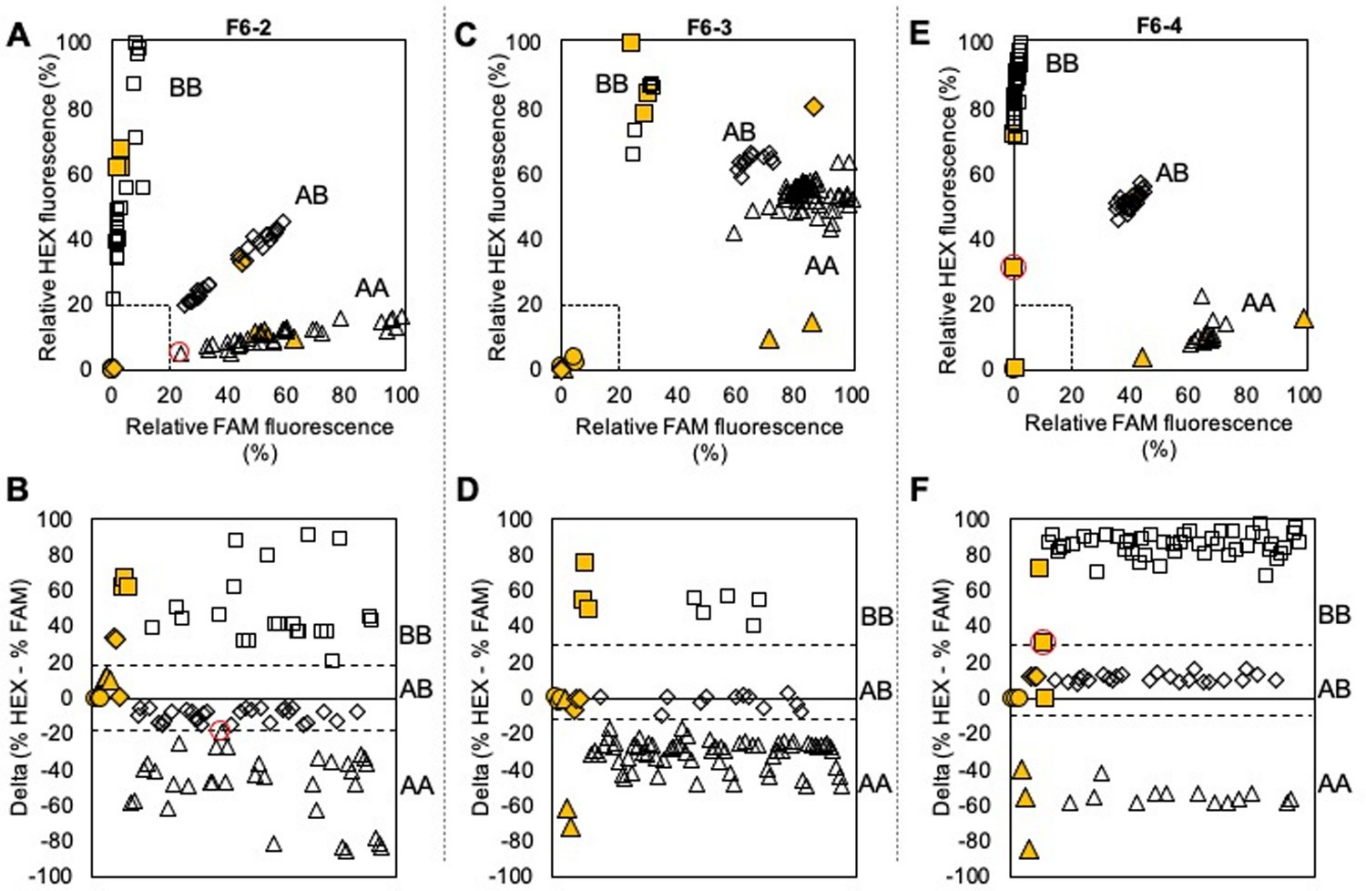
Results from KASP assay for SNPs on Ppe.XapF6 using clean DNA extraction and previously genotyped samples. Solid yellow shapes indicate positive controls, and empty shapes indicate unknowns, with the shape indicating the assigned genotype as follows: circle = no template/no amplification, triangle = AA, diamond = AB, square = BB. Dashed lines in plots in the top row indicate boundaries for no amplification (% fluorescence < ~20% for both fluorophores). Dashed lines in plots in the bottom row indicate delta (% HEX—% FAM) values separating heterozygous and homozygous genotypes, adjusted to reflect assay conditions. Points are automatically encircled in red when they are close enough to the dashed lines in the bottom row that manual inspection is advised. **A,B** = F6-2; **C,D** = F6-3; **E,F** = F6-4.

The KASP assays designed for each of the remaining seven SNPs successfully amplified and distinguished between AA, AB, and BB genotypes present in the validation germplasm (Table 1, Figs 1 and 2). Four clusters were observed for each KASP assay: high FAM fluorescence and low HEX fluorescence, high HEX fluorescence and low FAM fluorescence, intermediate FAM and HEX fluorescence, and ~0% FAM or HEX fluorescence. These clusters indicate genotypes of AA, AB, BB, and undetermined due to no amplification, respectively.

By collapsing FAM and HEX fluorescence values into the one-dimensional measurement, delta, we could easily assign samples to the appropriate genotype. We designed a user-friendly template spreadsheet to assign genotypes automatically based on default or user-specified parameters (S3 Table). No-template controls have delta values right at 0, while AB samples all fall slightly above or slightly below 0. All BB samples have delta values above a certain threshold, and all AA samples have delta values below a certain threshold. These thresholds can be set manually so that automatic genotype assignments reflect what is observed in the scatterplot of relative FAM and HEX fluorescence values. For example, for Ppe.XapF6-3, the AB and AA clusters were quite close together on the scatterplot (Fig 2C), but the threshold delta value was set so that the genotypes assigned by the delta plot (Fig 2D) corresponded to the clusters observed on the scatterplot (Fig 2C). On occasion, the delta value of a sample falls very close to the threshold; if it is within two units (an arbitrarily chosen, adjustable margin) of the threshold value the sample is flagged across all data plots to facilitate manual inspection. Across all seven assays, only two marginal calls occurred, one in the Ppe.XapF6-2 assay and one in the Ppe.XapF6-4 assay (Fig 2B and 2F). However, the positions of these samples on the relative fluorescence scatterplot (Fig 2A and 2E) clearly indicated that the original genotype assignment by the delta plot was correct.

In some cases, the positive controls failed, either by not amplifying (Ppe.XapF6-3) or by having an unexpected genotype (Ppe.XapF1-3, Fig 1E and 1F). As long as the four clusters (described above) were observed, genotypes could still be assigned. For Ppe.XapF1-3, the B allele was rare in germplasm used for validation, but a single BB genotype and two AB genotypes were identified by the KASP assay (Table 2). These individuals could be used as positive controls for future assays.

Thirty-two samples in the validation plate had known genotypes for *Xap*.*Pp*.*OC-1*.*2* and *Xap*.*Pp*.*OC-6*.*1*. Genotypes determined by the seven KASP assays matched the sequenced genotypes for all but two samples (breeding material BY07n3500x and cultivar Reliance), resulting in a 94% accuracy in allele assignment (Table 2, S5 Table). KASP-derived genotypes for the two exceptions changed allele assignments: ‘Reliance’ changed from I to unknown for Ppe.XapF1 and from homozygous R2 to R2|S for Ppe.XapF6 while BY07n3500x went from R1 to I for Ppe.XapF1.

### Endpoint assays

**Fig 3:**
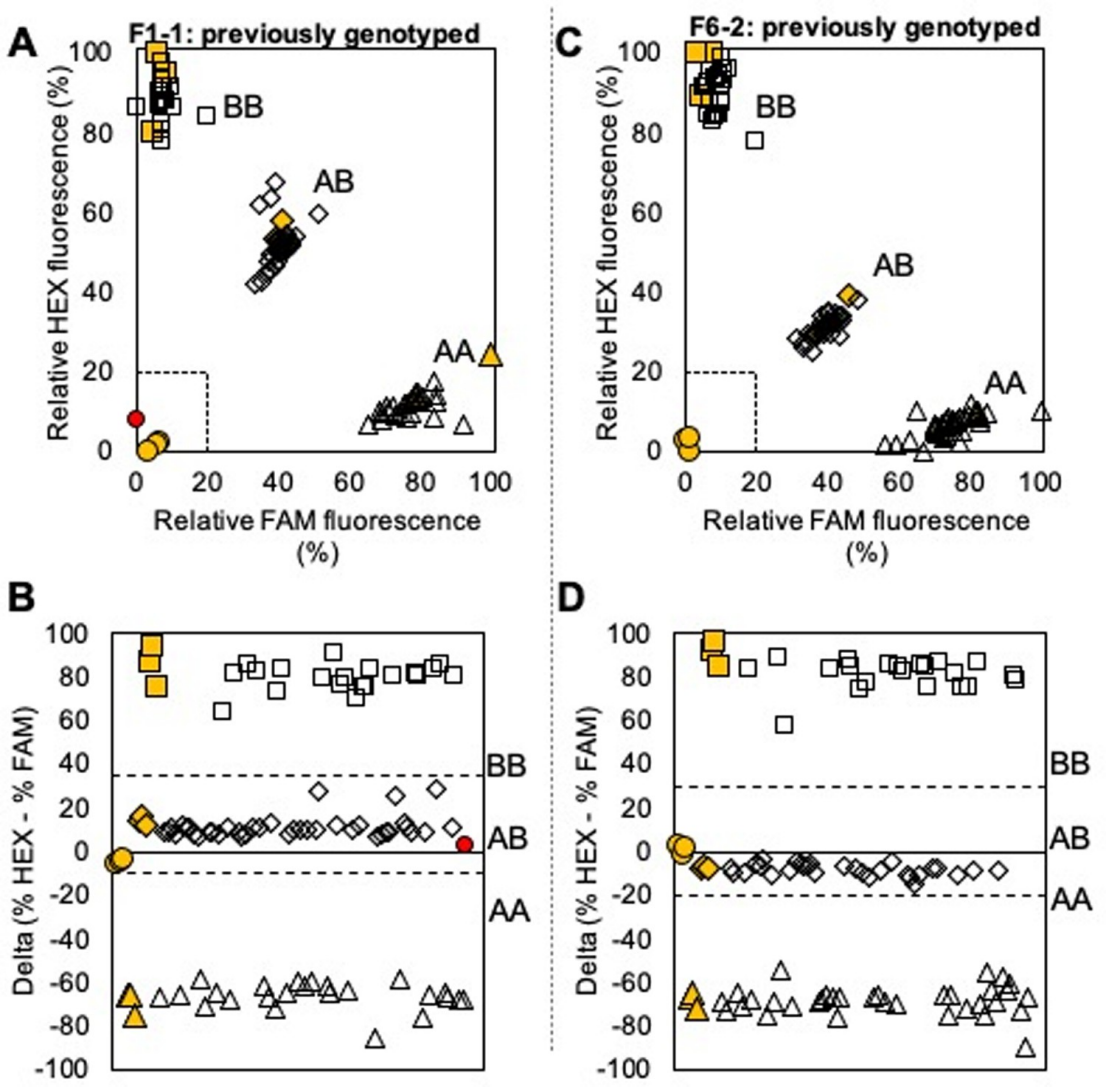
Results from KASP assays for two SNPs, Ppe.Xap F1-1 and Ppe.Xap F6-2, using endpoint PCR with previously genotyped samples. Solid yellow shapes indicate positive controls, solid red indicates an unknown that failed to amplify, and empty shapes indicate unknowns, with the shape indicating the assigned genotype as follows: circle = no template/no amplification, triangle = AA, diamond = AB, square = BB. Dashed lines in plots in the top row indicate boundaries for no amplification (% fluorescence < ~20% for both fluorophores). Dashed lines in plots in the bottom row indicate delta (% HEX—% FAM) values separating heterozygous and homozygous genotypes, adjusted to reflect assay conditions.

Real-time PCR ensures that an appropriate final cycle is chosen for genotype assignment, but limits assay throughput. Similarly, assaying all seven SNPs for complete allele assignment is time-consuming and not required to detect the S allele. To enable high throughput genotyping, endpoint PCR conditions were validated for the two KASP assays, Ppe.XapF1-1 and 6–2, where the A-coded allele (A|T nucleotides) is always associated with the S allele. For both assays, endpoint PCR performed similarly to real-time PCR, such that four distinct clusters were observed on fluorescence scatterplots and genotypes could be readily assigned (Fig 3). Amplification failed in 9% of samples and had to be repeated with different DNA concentration.

### Seedling genotyping and culling

**Fig 4:**
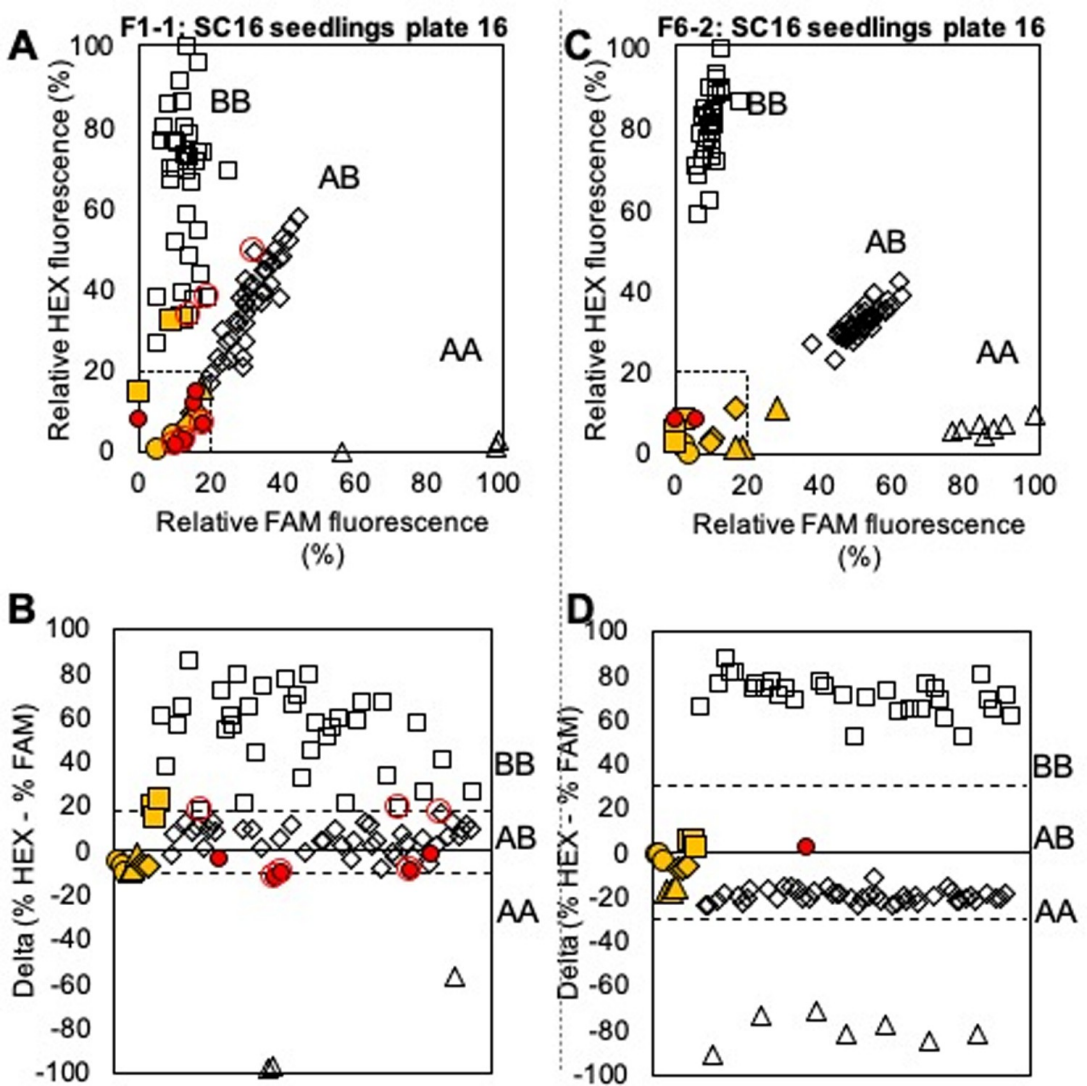
Results from KASP assay for two SNPs, Ppe.Xap F1-1 and Ppe.Xap F6-2, using endpoint PCR with crude DNA extracts from greenhouse-grown seedling samples. Solid yellow shapes indicate positive controls, solid red indicates an unknown that failed to amplify, and empty shapes indicate unknowns, with the shape indicating the assigned genotype as follows: circle = no template/no amplification, triangle = AA, diamond = AB, square = BB. Dashed lines in plots in the top row indicate boundaries for no amplification (% fluorescence < ~20% for both fluorophores). Dashed lines in plots in the bottom row indicate delta (% HEX—% FAM) values separating heterozygous and homozygous genotypes, adjusted to reflect assay conditions.

A total of 3,440 seedlings from 78 crosses generated in the CUPB across four years (2016–2020) were screened with KASP endpoint assays (Fig 4, S6 Table) for Ppe.XapF1-1 (S7 Table) and F6-2 (S8 Table) and a template spreadsheet was designed to simplify data management (S9 Table). After the user imports raw fluorescence data, which can be generated from multiple plates and SNP assays, SNP genotypes are automatically assigned and based on the genotype, seedlings to cull are flagged (S1 File). For the CUPB program, seedlings with ≥ 3 S alleles (i.e., homozygous S at one locus and heterozygous S at the other) were culled (S4 Table). Seedlings were also considered for culling, depending on parental genotype when F6-2 was homozygous S and F1-1 was homozygous for R (S4 Table), because *Xap*.*Pp*.*OC-6*.*1* is observed to contribute more to *Xap* tolerance than *Xap*.*Pp*.*OC-1*.*2*. The endpoint assay results flagged 1,588 seedlings for culling, reducing the number of planted seedlings by 46% (S6 Table). Precent of culled seedlings per cross ranged from 0–100% with an average of 44%. Culling of hybrids in crosses made in 2016 and 2017, that have already fruited in the field, averaged at 31% and was executed before start of the ripening season while culling of seedlings at the greenhouse stage (crosses 2018–2020) averaged at 51%.

## Discussion

In this research, we report new DNA tests that successfully identified fruit bacterial spot resistance alleles in breeding stock germplasm, including F_1_ individuals. Early screening for genetic resistance prior to field planting led to a dramatic decrease in the number of seedlings planted, as well as the number to be screened at maturity for productivity and fruit quality traits, saving a significant amount of money, labor, and time. This strategy also allows annual hybrid seedling production to go beyond the CUPB production target of ~3–4,000 hybrids, as only those hybrids with desired allele combinations at both *Xap* QTLs are planted. Nearly complete allele identification is possible by genotyping seven SNPs, although two alleles, R1 and “-”for the QTL on chromosome 6 are indistinguishable without genotyping an eighth SNP that does not work in KASP assays. The allele “-”(ABBB) on ch 6 was seen only in the heirloom cultivar Chinese Cling [18], among all U.S peach breeding germplasm [28] analyzed within the RosBREED project [19]. ‘Chinese Cling’ is an ancestor of all modern peach cultivars [29], and if one of its alleles is never observed in its descendants one could hypothesize that when passed to the next generation it resulted in an undesirable trait which caused it to be removed through selection by breeders. Additionally, the rare occurrence of this allele might also be explained by mutation occurring in the ‘Chinese Cling’ accession used as DNA source in the RosBREED project. The ‘Chinese Cling’, ancestor of all modern peach cultivars, was a seedling brought from China that was multiplied via seed, and many “copies” named ‘Chinese Cling’ exist in different germplasm collections.

In the development and validation of this DNA breeding tool, real-time and endpoint PCR results were in agreement, indicating that endpoint PCR can be adopted to increase throughput after optimizing reaction parameters with real-time PCR. Disagreement between the KASP and array [30] genotypes was observed in two accessions used in validation, resulting in 94% accuracy. In BY00n3500x the discrepancy was observed only for SNP F1-4, which reduced the predicted fruit response phenotype from R1 (array results) to I (KASP results). In ‘Reliance’, however, discrepancies were observed for 2 SNPs in each haploblock that completely changed the genotype and predicted fruit response to *Xap*. One explanation may be the trueness to type of the source of the ‘Reliance’ DNA. The array genotypes obtained in the RosBREED project were based on ‘Reliance’ from the *Prunus* germplasm collection at the National Clonal Germplasm Repository (NCGR) [31] in Davis, CA. The DNA for KASP assay validation in this study was extracted from trees in the CUPB *Prunus* collection. Obtaining genome sequences of these accessions, including the ‘Reliance’ from NCGR, is needed to verify the genotype.

The fruit tolerance prediction based on haplotyping with KASP assays disagreed with reported responses in four accessions (‘Flavortop’, ‘Harvester’, ‘SC08-13-001’ and ‘Winblo’), with lower tolerance predicted for three and higher tolerance for one accession than reported in the literature (Table 2). Since fruit and foliar responses are independently inherited in peach [8, 15], the discrepancies may arise because the Ppe.XapF assays were developed for the peach fruit response QTLs, while the *Xap* responses reported in literature are provided as a single phenotype combining both fruit and foliar responses [12]. Moreover, the phenotypes associated with *Xap*.*Pp*.*OC-1*.*2* and *Xap*.*Pp*.*OC-6*.*1* alleles [18], determined from a mapping study [15] and breeders’ records [19], suggest a possible epistatic effect of the two QTLs, were alleles on ch 6 contribute more towards fruit tolerance to *Xap* than alleles on ch 1, however alleles from both QTLs are important for resistant response. Therefore, culling decisions in the CUPB program, while based on alleles from both ch 1 and 6, are heavily influenced by the presence of S alleles on ch 6.

In our previous study [18] we profiled relevant U.S. peach breeding germplasm [28, 32] for presence and frequency of alleles associated with *Xap* fruit response. Information about alleles associated with fruit *Xap* responses for important founders, breeding parents and recently released peach cultivars relevant to the U.S. modern peach breeding was generated within the RosBREED project [19] and is freely available at www.rosbreed.org. This information is used in cross design in the CUPB program with a goal to achieve resistant *Xap* fruit phenotype (S6 Table). The two markers we chose for high throughput endpoint analysis to guide seedling culling decisions in the CUPB program could be relevant for any peach breeding program in North America and Europe, due to the shared ancestry of peach breeding germplasm. All seven KASP assays, or just the two we selected, could be used to profile material in any peach breeding program with high certainty of predicting the fruit response to bacterial spot. Most peach breeding programs in the U.S. and Europe have already obtained genotypes of their material using the peach 9K SNP array and can profile important parents in their breeding programs for alleles in these two *Xap* QTLs. In addition to the seedling screening, the Ppe.XapF assays developed in this study could also be used to quickly and cost effectively determine XapF genotypes/phenotypes of potential breeding parents.

A high-throughput workflow was established, using a crude but rapid DNA extraction method, a robotic liquid handler for assay setup in 96-well plates, and endpoint PCR assays followed by bulk plate reading on a real-time machine. The stability of crude DNA is limited to a few days at +4°C or longer at -20°C [26]. The post-DNA extraction workflow can easily process 32 96-well plates per typical workday with a single technician with very low cost. The workflow is limited only by the number of endpoint PCR units available, as the approximate time to run an endpoint PCR assay is less than 2 hours and reading the result on a real-time unit takes less than 3 minutes. Using automation to dilute samples and prepare DNA for PCR reactions eliminated human pipetting errors and ensured reproducibility of results.

Although the software accompanying real-time units produces genotype calls, the calls can often disagree with the user’s perception of what the call should be and are difficult to adjust within the software. Using the delta method, especially as incorporated in the template spreadsheet described and provided herein, the user can easily change genotype assignments and determine genotype-to-well associations unambiguously. The user can also adjust the sensitivity of the method to specific criteria.

Assay results from crude DNA were more variable than those from high-quality DNA: more samples failed to amplify, and samples at the upper or lower boundaries of the AB genotype occurred more frequently. Problems with sample amplification due to low DNA concentration occurred in ~9% of samples, and samples that did not amplify generally failed in several assays, a clear indication that DNA was too dilute. This led to repeating 379 assays with crude DNA at a higher concentration (5× instead of 40× dilution), which was sufficient to solve the problem. Crude DNA extracted from leaf tissue collected from seedlings already planted in the field (1,774 hybrid seedlings from crossing years 2016–2017) had expected concentrations of 180–400 ng μL^-1^ and successfully amplified. However, crude DNA extracted from 1,666 seedlings in the greenhouse from crossing seasons 2018–2020 had variable concentrations directly associated with the age of plantlets, as younger plantlets had lower DNA concentration and required less dilution for successful PCR. Adequate dilution of crude DNA is essential for successful PCR, and a balance must be maintained between diluting away inhibitors and maintaining appropriate DNA concentration for amplification within 40 cycles, as was suggested by Noh *et al*. [26] for strawberry.

## Conclusions

Ppe.XapF KASP assays for SNPs associated with peach fruit *Xap* resistance were developed and validated on crude DNA extractions of individuals with known and unknown genotypes. These assays were highly accurate in identifying the correct genotype for 94% of samples with known genotypes. Of the seven assays developed, just two–one for ch 1 and one for ch 6 –are enough to reveal susceptible alleles. End-users have control over genotype assignment by using the delta method, which also highlights any problematic samples or assays. These assays provide a rapid, cheap, accessible method to cull seedlings with undesirable genotypes before field planting, and to flag seedlings with desired genotypes for further genotyping and phenotyping. They can also be used to profile favorable parents in peach breeding programs and design crosses based on the *Xap* fruit tolerance.

## Supporting information

S1 Table Annealing temperatures (Tm, °C) and primer sequences for each assay, including HEX (GAAGGTCGGAGTCAACGGATT) and FAM (GAAGGTGACCAAGTTCATGCT) tag sequences. The final lowercase nucleotide of each forward primer sequence is the assayed SNP. “Tag” indicates which fluorophore is used: “F” = FAM, “H” = HEX, “N” = none, for the reverse primer.
(DOCX)

S2 Table Accessions used as controls for each KASP assay. (DOCX)

S3 Table Template spreadsheet for analyzing one SNP. (XLSX)

S4 Table Parameters for keeping or culling seedlings based on Ppe.XapF1-1 and Ppe.XapF6-2 KASP assays in the Clemson University peach breeding program. (DOCX)

S5 Table Peach material used in development and validation of the Ppe.XapF DNA test including KASP and peach 9K SNP array genotypes. S = Susceptible, I = Intermediate, R1 = Resistant-1, R2 = Resistant-2,— = unknown Xap response allele.
(XLSX)

S6 Table Origin and number of seedlings per cross included in the study. Parental Xap genotypes on chromosomes 1 and 6 are separate with |, respectively. Ppe.Xap F1-1 and F6-2 endpoint PCR results.
(XLSX)

S7 Table KASP results for Ppe.XapF1-1 assays on seedlings in the Clemson peach breeding program across four years, resulting from using the spreadsheets available as S9 Table. (XLSX)

S8 Table KASP results for Ppe.XapF6-2 assays on seedlings in the Clemson peach breeding program across four years, resulting from using the spreadsheets available as S9 Table. (XLSX)

S9 Table Template spreadsheet for analyzing SNPs Ppe.XapF1-1 and Ppe.XapF6-2 across many plates of material. (XLSX)

S1 File Template file with detailed instructions on KASP data analyses for 20 plates (20) for Ppe.XapF1-1 and 6–2 assays. (XLSX)
