# Supplementary material for: Ppe.XapF: High throughput KASP assays to identify fruit response to Xanthomonas arboricola pv. pruni (Xap) in peach
Source: PLoS One. 2022 Feb 25;17(2):e0264543. doi: 10.1371/journal.pone.0264543 (PMC8880879; doi:10.1371/journal.pone.0264543)
Supplement: S1 Table — The final lowercase nucleotide of each forward primer sequence is the assayed SNP. “Tag” indicates which fluorophore is used: “F” = FAM, “H” = HEX, “N” = none, for the reverse primer. (DOCX) [file pone.0264543.s001.docx]

Supplementary Table 1. Annealing temperatures (Tm, ^o^C) and primer sequences for each assay, including HEX (GAAGGTCGGAGTCAACGGATT) and FAM (GAAGGTGACCAAGTTCATGCT) tag sequences. The final lowercase nucleotide of each forward primer sequence is the assayed SNP. “Tag” indicates which fluorophore is used: “F” = FAM, “H” = HEX, “N” = none, for the reverse primer.

| SNP | Tm | Tag | Primer sequence (5' 🡪 3') |
| --- | --- | --- | --- |
| F1-1 | 55 | F | GAAGGTGACCAAGTTCATGCTCATTACCTTTGATCCATTCCAAAGACa |
|  |  | H | GAAGGTCGGAGTCAACGGATTCATTACCTTTGATCCATTCCAAAGACg |
|  |  | N | GCAATTTCTGAACTTAATTTGCGTCATAGC |
| F1-2 | 55 | F | GAAGGTGACCAAGTTCATGCTAACGACATGACGTAGTGGTTGAGTTa |
|  |  | H | GAAGGTCGGAGTCAACGGATTAACGACATGACGTAGTGGTTGAGTTg |
|  |  | N | GGAGATAATCAATGACTTCCAATGTGGC |
| F1-3 | 58 | F | GAAGGTGACCAAGTTCATGCTTAGAGAAACAGCTGCCATAACCTc |
|  |  | H | GAAGGTCGGAGTCAACGGATTTAGAGAAACAGCTGCCATAACCTt |
|  |  | N | CTTTACTTTCATGCGGCTCATAACCA |
| F1-4 | 55 | F | GAAGGTGACCAAGTTCATGCTAGAGGAAGACATACTTTATCAATCCATCCAAa |
|  |  | H | GAAGGTCGGAGTCAACGGATTAGAGGAAGACATACTTTATCAATCCATCCAAg |
|  |  | N | TGTAGGGAATGTGCTCTTTCTGTTGAAG |
| F6-2 | 55 | F | GAAGGTGACCAAGTTCATGCTCAAAGTTGTACCAGGTCATCCTTTTCAATAt |
|  |  | H | GAAGGTCGGAGTCAACGGATTCAAAGTTGTACCAGGTCATCCTTTTCAATAc |
|  |  | N | GAAACAATGCTTTGCATATTGCTCAATGTT |
| F6-3 | 59 | F | GAAGGTGACCAAGTTCATGCTTATTTGCTCGTACTCGATGATGTTTGGAAt |
|  |  | H | GAAGGTCGGAGTCAACGGATTTATTTGCTCGTACTCGATGATGTTTGGAAc |
|  |  | N | GTGTATCTTTAACACTTGACAAACAACTCGTC |
| F6-4 | 55 | F | GAAGGTGACCAAGTTCATGCTTAATTGGCAGAGAGATGGGAGGCt |
|  |  | H | GAAGGTCGGAGTCAACGGATTTAATTGGCAGAGAGATGGGAGGCc |
|  |  | N | GGAAACTCGAAGCTATTCCAATCACA |
