# Supplementary material for: Ppe.XapF: High throughput KASP assays to identify fruit response to Xanthomonas arboricola pv. pruni (Xap) in peach
Source: PLoS One. 2022 Feb 25;17(2):e0264543. doi: 10.1371/journal.pone.0264543 (PMC8880879; doi:10.1371/journal.pone.0264543)
Supplement: S2 Table — (DOCX) [file pone.0264543.s002.docx]

Supplementary Table 2. Accessions used as controls for each KASP assay

| KASP Assay | Controls | | |
| --- | --- | --- | --- |
|  | AA | AB | BB |
| Ppe.XapF1-1 | SC08_16_005 | Clayton | Loring |
| Ppe.XapF1-2 | SC08_16_005 | Clayton | Loring |
| Ppe.XapF1-3 | Loring | SC08_16_005 | Reliance |
| Ppe.XapF1-4 | Loring | Clayton | SC08_16_005 |
| Ppe.XapF6-2 | SC08_16_005 | Redhaven | Loring |
| Ppe.XapF6-3 | Loring | Clayton | BY00P4945 |
| Ppe.XapF6-4 | Loring | Redhaven | BY00P4945 |
