# Supplementary material for: Ppe.XapF: High throughput KASP assays to identify fruit response to Xanthomonas arboricola pv. pruni (Xap) in peach
Source: PLoS One. 2022 Feb 25;17(2):e0264543. doi: 10.1371/journal.pone.0264543 (PMC8880879; doi:10.1371/journal.pone.0264543)
Supplement: S4 Table — (DOCX) [file pone.0264543.s004.docx]

Supplementary Table 4. Parameters for keeping or culling seedlings based on Ppe.XapF1-1 and Ppe.XapF6-2 KASP assays in the Clemson University peach breeding program.

| F1-1 genotype | F6-2 genotype | A dose | B dose | Seedling fate |
| --- | --- | --- | --- | --- |
| AA | BB | 2 | 2 | keep |
| AB | AB | 2 | 2 | keep |
| AB | BB | 1 | 3 | keep |
| BB | AB | 1 | 3 | keep |
| BB | BB | 0 | 4 | keep |
| AA | AA | 4 | 0 | cull |
| AA | AB | 3 | 1 | cull |
| AB | AA | 3 | 1 | cull |
| BB | AA | 2 | 2 | cull |
